# Supplementary material for: HIHISIV: a database of gene expression in HIV and SIV host immune response
Source: BMC Bioinformatics. 2024 Mar 22;25:125. doi: 10.1186/s12859-024-05740-7 (PMC10958971; doi:10.1186/s12859-024-05740-7)
Supplement: Supplementary file 1 — Additional file 1 includes a table of the datasets/experiments in the current HIHISIV database (v2.0). [file 12859_2024_5740_MOESM1_ESM.docx]

Supplementary Material 1

| **geo_accession** | **experiment_id (normalized matrix download)** | **reference_factor_name** | **test_factor_name** | **platform_id** | **num_samples** | **num_samples_per_group (phenodata download)** | **species** | **sp_ncbi_id** |
| --- | --- | --- | --- | --- | --- | --- | --- | --- |
| [GSE6740](https://www.ncbi.nlm.nih.gov/geo/query/acc.cgi?acc=GSE6740) | [GSE6740_d1](https://github.com/hihisiv/matrix_normalized/blob/main/GSE6740_d1-MatrixNormalized.csv) | CD4+ | CD8+ | GPL96 | 10 | [5 CD4 acute; 5 CD8 acute](https://github.com/hihisiv/phenodata/blob/main/GSE6740_d1.csv) | *Homo sapiens* | 9606 |
| [GSE6740](https://www.ncbi.nlm.nih.gov/geo/query/acc.cgi?acc=GSE6740) | [GSE6740_d2](https://github.com/hihisiv/matrix_normalized/blob/main/GSE6740_d2-MatrixNormalized.csv) | CD4+ | CD8+ | GPL96 | 10 | [5 CD4 chronic; 5 CD8 chronic](https://github.com/hihisiv/phenodata/blob/main/GSE6740_d2.csv) | *Homo sapiens* | 9606 |
| [GSE6740](https://www.ncbi.nlm.nih.gov/geo/query/acc.cgi?acc=GSE6740) | [GSE6740_d3](https://github.com/hihisiv/matrix_normalized/blob/main/GSE6740_d3-MatrixNormalized.csv) | CD4+ | CD8+ | GPL96 | 10 | [5 CD4 uninfected; 5 CD8 uninfected](https://github.com/hihisiv/phenodata/blob/main/GSE6740_d3.csv) | *Homo sapiens* | 9606 |
| [GSE6740](https://www.ncbi.nlm.nih.gov/geo/query/acc.cgi?acc=GSE6740) | [GSE6740_d4](https://github.com/hihisiv/matrix_normalized/blob/main/GSE6740_d4-MatrixNormalized.csv) | CD4+ | CD8+ | GPL96 | 10 | [5 CD4 non-progressor; 5 CD8 non-progressor](https://github.com/hihisiv/phenodata/blob/main/GSE6740_d4.csv) | *Homo sapiens* | 9606 |
| [GSE6740](https://www.ncbi.nlm.nih.gov/geo/query/acc.cgi?acc=GSE6740) | [GSE6740_d5](https://github.com/hihisiv/matrix_normalized/blob/main/GSE6740_d5-MatrixNormalized.csv) | acute infection | chronic infection | GPL96 | 10 | [5 CD4 acute; 5 CD4 chronic](https://github.com/hihisiv/phenodata/blob/main/GSE6740_d5.csv) | *Homo sapiens* | 9606 |
| [GSE6740](https://www.ncbi.nlm.nih.gov/geo/query/acc.cgi?acc=GSE6740) | [GSE6740_d6](https://github.com/hihisiv/matrix_normalized/blob/main/GSE6740_d6-MatrixNormalized.csv) | uninfected | acute infection | GPL96 | 10 | [5 CD4 uninfected; 5 CD4 acute](https://github.com/hihisiv/phenodata/blob/main/GSE6740_d6.csv) | *Homo sapiens* | 9606 |
| [GSE6740](https://www.ncbi.nlm.nih.gov/geo/query/acc.cgi?acc=GSE6740) | [GSE6740_d7](https://github.com/hihisiv/matrix_normalized/blob/main/GSE6740_d7-MatrixNormalized.csv) | non-progressor | acute infection | GPL96 | 10 | [5 CD4 non-progressor; 5 CD4 acute](https://github.com/hihisiv/phenodata/blob/main/GSE6740_d7.csv) | *Homo sapiens* | 9606 |
| [GSE6740](https://www.ncbi.nlm.nih.gov/geo/query/acc.cgi?acc=GSE6740) | [GSE6740_d8](https://github.com/hihisiv/matrix_normalized/blob/main/GSE6740_d8-MatrixNormalized.csv) | non-progressor | chronic infection | GPL96 | 10 | [5 CD4 non-progressor; 5 CD4 chronic](https://github.com/hihisiv/phenodata/blob/main/GSE6740_d8.csv) | *Homo sapiens* | 9606 |
| [GSE6740](https://www.ncbi.nlm.nih.gov/geo/query/acc.cgi?acc=GSE6740) | [GSE6740_d9](https://github.com/hihisiv/matrix_normalized/blob/main/GSE6740_d9-MatrixNormalized.csv) | uninfected | chronic infection | GPL96 | 10 | [5 CD4 uninfected; 5 CD4 chronic](https://github.com/hihisiv/phenodata/blob/main/GSE6740_d9.csv) | *Homo sapiens* | 9606 |
| [GSE6740](https://www.ncbi.nlm.nih.gov/geo/query/acc.cgi?acc=GSE6740) | [GSE6740_d10](https://github.com/hihisiv/matrix_normalized/blob/main/GSE6740_d10-MatrixNormalized.csv) | uninfected | non-progressor | GPL96 | 10 | [5 CD4 uninfected; 5 CD4 non-progressor](https://github.com/hihisiv/phenodata/blob/main/GSE6740_d10.csv) | *Homo sapiens* | 9606 |
| [GSE6740](https://www.ncbi.nlm.nih.gov/geo/query/acc.cgi?acc=GSE6740) | [GSE6740_d11](https://github.com/hihisiv/matrix_normalized/blob/main/GSE6740_d11-MatrixNormalized.csv) | acute infection | chronic infection | GPL96 | 10 | [5 CD8 acute; 5 CD8 chronic](https://github.com/hihisiv/phenodata/blob/main/GSE6740_d11.csv) | *Homo sapiens* | 9606 |
| [GSE6740](https://www.ncbi.nlm.nih.gov/geo/query/acc.cgi?acc=GSE6740) | [GSE6740_d12](https://github.com/hihisiv/matrix_normalized/blob/main/GSE6740_d12-MatrixNormalized.csv) | uninfected | acute infection | GPL96 | 10 | [5 CD8 uninfected; 5 CD8 acute](https://github.com/hihisiv/phenodata/blob/main/GSE6740_d12.csv) | *Homo sapiens* | 9606 |
| [GSE6740](https://www.ncbi.nlm.nih.gov/geo/query/acc.cgi?acc=GSE6740) | [GSE6740_d13](https://github.com/hihisiv/matrix_normalized/blob/main/GSE6740_d13-MatrixNormalized.csv) | non-progressor | acute infection | GPL96 | 10 | [5 CD8 non-progressor; 5 CD8 acute](https://github.com/hihisiv/phenodata/blob/main/GSE6740_d13.csv) | *Homo sapiens* | 9606 |
| [GSE6740](https://www.ncbi.nlm.nih.gov/geo/query/acc.cgi?acc=GSE6740) | [GSE6740_d14](https://github.com/hihisiv/matrix_normalized/blob/main/GSE6740_d14-MatrixNormalized.csv) | non-progressor | chronic infection | GPL96 | 10 | [5 CD8 non-progressor; 5 CD8 chronic](https://github.com/hihisiv/phenodata/blob/main/GSE6740_d14.csv) | *Homo sapiens* | 9606 |
| [GSE6740](https://www.ncbi.nlm.nih.gov/geo/query/acc.cgi?acc=GSE6740) | [GSE6740_d15](https://github.com/hihisiv/matrix_normalized/blob/main/GSE6740_d15-MatrixNormalized.csv) | uninfected | chronic infection | GPL96 | 10 | [5 CD8 uninfected; 5 CD8 chronic](https://github.com/hihisiv/phenodata/blob/main/GSE6740_d15.csv) | *Homo sapiens* | 9606 |
| [GSE6740](https://www.ncbi.nlm.nih.gov/geo/query/acc.cgi?acc=GSE6740) | [GSE6740_d16](https://github.com/hihisiv/matrix_normalized/blob/main/GSE6740_d16-MatrixNormalized.csv) | uninfected | non-progressor | GPL96 | 10 | [5 CD8 uninfected; 5 CD8 non-progressor](https://github.com/hihisiv/phenodata/blob/main/GSE6740_d16.csv) | *Homo sapiens* | 9606 |
| [GSE6740](https://www.ncbi.nlm.nih.gov/geo/query/acc.cgi?acc=GSE6740) | [GSE6740_d17](https://github.com/hihisiv/matrix_normalized/blob/main/GSE6740_d17-MatrixNormalized.csv) | acute infection | chronic infection | GPL96 | 20 | [10 acute; 10 chronic](https://github.com/hihisiv/phenodata/blob/main/GSE6740_d17.csv) | *Homo sapiens* | 9606 |
| [GSE6740](https://www.ncbi.nlm.nih.gov/geo/query/acc.cgi?acc=GSE6740) | [GSE6740_d18](https://github.com/hihisiv/matrix_normalized/blob/main/GSE6740_d18-MatrixNormalized.csv) | uninfected | acute infection | GPL96 | 20 | [10 uninfected; 10 acute](https://github.com/hihisiv/phenodata/blob/main/GSE6740_d18.csv) | *Homo sapiens* | 9606 |
| [GSE6740](https://www.ncbi.nlm.nih.gov/geo/query/acc.cgi?acc=GSE6740) | [GSE6740_d19](https://github.com/hihisiv/matrix_normalized/blob/main/GSE6740_d19-MatrixNormalized.csv) | uninfected | chronic infection | GPL96 | 20 | [10 uninfected; 10 chronic](https://github.com/hihisiv/phenodata/blob/main/GSE6740_d19.csv) | *Homo sapiens* | 9606 |
| [GSE6740](https://www.ncbi.nlm.nih.gov/geo/query/acc.cgi?acc=GSE6740) | [GSE6740_d20](https://github.com/hihisiv/matrix_normalized/blob/main/GSE6740_d20-MatrixNormalized.csv) | uninfected | non-progressor | GPL96 | 20 | [10 uninfected; 10 non-progressor](https://github.com/hihisiv/phenodata/blob/main/GSE6740_d20.csv) | *Homo sapiens* | 9606 |
| [GSE6740](https://www.ncbi.nlm.nih.gov/geo/query/acc.cgi?acc=GSE6740) | [GSE6740_d21](https://github.com/hihisiv/matrix_normalized/blob/main/GSE6740_d21-MatrixNormalized.csv) | non-progressor | acute infection | GPL96 | 20 | [10 non-progressor; 10 acute](https://github.com/hihisiv/phenodata/blob/main/GSE6740_d21.csv) | *Homo sapiens* | 9606 |
| [GSE6740](https://www.ncbi.nlm.nih.gov/geo/query/acc.cgi?acc=GSE6740) | [GSE6740_d22](https://github.com/hihisiv/matrix_normalized/blob/main/GSE6740_d22-MatrixNormalized.csv) | non-progressor | chronic infection | GPL96 | 20 | [10 non-progressor; 10 chronic](https://github.com/hihisiv/phenodata/blob/main/GSE6740_d22.csv) | *Homo sapiens* | 9606 |
| [GSE7157](https://www.ncbi.nlm.nih.gov/geo/query/acc.cgi?acc=GSE7157) | [GSE7157_d1](https://github.com/hihisiv/matrix_normalized/blob/main/GSE7157_d1-MatrixNormalized.csv) | uninfected | acute infection | GPL3535 | 14 | [7 at baseline prior to infection; 7 week 2](https://github.com/hihisiv/phenodata/blob/main/GSE7157_d1.csv) | Macaca fascicularis | 9541 |
| [GSE7157](https://www.ncbi.nlm.nih.gov/geo/query/acc.cgi?acc=GSE7157) | [GSE7157_d2](https://github.com/hihisiv/matrix_normalized/blob/main/GSE7157_d2-MatrixNormalized.csv) | uninfected | chronic infection | GPL3535 | 14 | [7 at baseline prior to infection; 7 week 20](https://github.com/hihisiv/phenodata/blob/main/GSE7157_d2.csv) | Macaca fascicularis | 9541 |
| [GSE7157](https://www.ncbi.nlm.nih.gov/geo/query/acc.cgi?acc=GSE7157) | [GSE7157_d3](https://github.com/hihisiv/matrix_normalized/blob/main/GSE7157_d3-MatrixNormalized.csv) | acute infection | chronic infection | GPL3535 | 14 | [7 week 2; 7 week 20](https://github.com/hihisiv/phenodata/blob/main/GSE7157_d3.csv) | Macaca fascicularis | 9541 |
| [GSE13824](https://www.ncbi.nlm.nih.gov/geo/query/acc.cgi?acc=GSE13824) | [GSE13824_d1](https://github.com/hihisiv/matrix_normalized/blob/main/GSE13824_d1-MatrixNormalized.csv) | uninfected | infected | GPL3535 | 36 | [9 uninfected (18 samples) and 9 infected (18 samples) RMs](https://github.com/hihisiv/phenodata/blob/main/GSE13824_d1.csv) | *Macaca mulatta* | 9544 |
| [GSE16147](https://www.ncbi.nlm.nih.gov/geo/query/acc.cgi?acc=GSE16147) | [GSE16147_d1](https://github.com/hihisiv/matrix_normalized/blob/main/GSE16147_d1-MatrixNormalized.csv) | acute infection; natural host | acute infection; non_natural host | GPL3535 | 47 | [25 SM; 22 RM](https://github.com/hihisiv/phenodata/blob/main/GSE16147_d1.csv) | *Cercocebus atys*; *Macaca mulatta* | 9531; 9544 |
| [GSE16147](https://www.ncbi.nlm.nih.gov/geo/query/acc.cgi?acc=GSE16147) | [GSE16147_d2](https://github.com/hihisiv/matrix_normalized/blob/main/GSE16147_d2-MatrixNormalized.csv) | uninfected | acute infection | GPL3535 | 28 | [3 SM uninfected; 25 SM acute](https://github.com/hihisiv/phenodata/blob/main/GSE16147_d2.csv) | *Cercocebus atys* | 9531 |
| [GSE16147](https://www.ncbi.nlm.nih.gov/geo/query/acc.cgi?acc=GSE16147) | [GSE16147_d3](https://github.com/hihisiv/matrix_normalized/blob/main/GSE16147_d3-MatrixNormalized.csv) | uninfected | chronic infection | GPL3535 | 8 | [3 SM uninfected; 5 SM chronic](https://github.com/hihisiv/phenodata/blob/main/GSE16147_d3.csv) | *Cercocebus atys* | 9531 |
| [GSE16147](https://www.ncbi.nlm.nih.gov/geo/query/acc.cgi?acc=GSE16147) | [GSE16147_d4](https://github.com/hihisiv/matrix_normalized/blob/main/GSE16147_d4-MatrixNormalized.csv) | acute infection | chronic infection | GPL3535 | 30 | [25 SM acute; 5 SM chronic](https://github.com/hihisiv/phenodata/blob/main/GSE16147_d4.csv) | *Cercocebus atys* | 9531 |
| [GSE16147](https://www.ncbi.nlm.nih.gov/geo/query/acc.cgi?acc=GSE16147) | [GSE16147_d5](https://github.com/hihisiv/matrix_normalized/blob/main/GSE16147_d5-MatrixNormalized.csv) | uninfected | acute infection | GPL3535 | 30 | [8 RM uninfected; 22 RM acute](https://github.com/hihisiv/phenodata/blob/main/GSE16147_d5.csv) | *Macaca mulatta* | 9544 |
| [GSE17626](https://www.ncbi.nlm.nih.gov/geo/query/acc.cgi?acc=GSE17626) | [GSE17626_d1](https://github.com/hihisiv/matrix_normalized/blob/main/GSE17626_d1-MatrixNormalized.csv) | uninfected | acute infection | GPL3535 | 16 | [4 RMs without SIV infection (8 samples) and 4 RMs SIVmac239-infected (14 dpi; 8 samples)](https://github.com/hihisiv/phenodata/blob/main/GSE17626_d1.csv) | *Macaca mulatta* | 9544 |
| [GSE17626](https://www.ncbi.nlm.nih.gov/geo/query/acc.cgi?acc=GSE17626) | [GSE17626_d2](https://github.com/hihisiv/matrix_normalized/blob/main/GSE17626_d2-MatrixNormalized.csv) | uninfected | acute infection | GPL3535 | 12 | [2 SMs prior to infection (FRs and Fuv; 4 samples); 2 SMs at 14 dpi (FRs and FWv; 4 samples); 2 SMs at 30 dpi (Fuv and FWv; 4 samples)](https://github.com/hihisiv/phenodata/blob/main/GSE17626_d2.csv) | *Cercocebus atys* | 9531 |
| [GSE17626](https://www.ncbi.nlm.nih.gov/geo/query/acc.cgi?acc=GSE17626) | [GSE17626_d3](https://github.com/hihisiv/matrix_normalized/blob/main/GSE17626_d3-MatrixNormalized.csv) | uninfected; natural host | uninfected; acute | GPL3535 | 12 | [4 SM uninfected; 8 RM uninfected](https://github.com/hihisiv/phenodata/blob/main/GSE17626_d3.csv) | *Cercocebus atys*; *Macaca mulatta* | 9531; 9544 |
| [GSE17626](https://www.ncbi.nlm.nih.gov/geo/query/acc.cgi?acc=GSE17626) | [GSE17626_d4](https://github.com/hihisiv/matrix_normalized/blob/main/GSE17626_d4-MatrixNormalized.csv) | acute infection; natural host | acute infection; non_natural host | GPL3535 | 16 | [8 SM acute; 8 RM acute](https://github.com/hihisiv/phenodata/blob/main/GSE17626_d4.csv) | *Cercocebus atys*; *Macaca mulatta* | 9531; 9544 |
| [GSE24081](https://www.ncbi.nlm.nih.gov/geo/query/acc.cgi?acc=GSE24081) | [GSE24081_d1](https://github.com/hihisiv/matrix_normalized/blob/main/GSE24081_d1-MatrixNormalized.csv) | controller | chronic infection | GPL3921 | 42 | [24 HIV controllers; 18 chronic progressors](https://github.com/hihisiv/phenodata/blob/main/GSE24081_d1.csv) | *Homo sapiens* | 9606 |
| [GSE29980](https://www.ncbi.nlm.nih.gov/geo/query/acc.cgi?acc=GSE29980) | [GSE29980_d1](https://github.com/hihisiv/matrix_normalized/blob/main/GSE29980_d1-MatrixNormalized.csv) | uninfected | acute infection | GPL3535 | 8 | [4 RM uninfected; 4 RM acute](https://github.com/hihisiv/phenodata/blob/main/GSE29980_d1.csv) | *Macaca mulatta* | 9544 |
| [GSE29980](https://www.ncbi.nlm.nih.gov/geo/query/acc.cgi?acc=GSE29980) | [GSE29980_d2](https://github.com/hihisiv/matrix_normalized/blob/main/GSE29980_d2-MatrixNormalized.csv) | uninfected | chronic infection | GPL3535 | 8 | [4 SM uninfected; 4 SM chronic](https://github.com/hihisiv/phenodata/blob/main/GSE29980_d2.csv) |  | 9531 |
| [GSE34300](https://www.ncbi.nlm.nih.gov/geo/query/acc.cgi?acc=GSE34300) | [GSE34300_d1](https://github.com/hihisiv/matrix_normalized/blob/main/GSE34300_d1-MatrixNormalized.csv) | uninfected | acute infection | GPL3535 | 6 | [3 uninfected; 3 acute](https://github.com/hihisiv/phenodata/blob/main/GSE34300_d1.csv) | *Macaca mulatta* | 9544 |
| [GSE34300](https://www.ncbi.nlm.nih.gov/geo/query/acc.cgi?acc=GSE34300) | [GSE34300_d2](https://github.com/hihisiv/matrix_normalized/blob/main/GSE34300_d2-MatrixNormalized.csv) | uninfected | chronic infection | GPL3535 | 6 | [3 uninfected; 3 chronic](https://github.com/hihisiv/phenodata/blob/main/GSE34300_d2.csv) | *Macaca mulatta* | 9544 |
| [GSE34300](https://www.ncbi.nlm.nih.gov/geo/query/acc.cgi?acc=GSE34300) | [GSE34300_d3](https://github.com/hihisiv/matrix_normalized/blob/main/GSE34300_d3-MatrixNormalized.csv) | acute | chronic infection | GPL3535 | 6 | [3 acute; 3 chronic](https://github.com/hihisiv/phenodata/blob/main/GSE34300_d3.csv) | *Macaca mulatta* | 9544 |
| [GSE51436](https://www.ncbi.nlm.nih.gov/geo/query/acc.cgi?acc=GSE51436) | [GSE51436_d1](https://github.com/hihisiv/matrix_normalized/blob/main/GSE51436_d1-MatrixNormalized.csv) | uninfected | infected | GPL3535 | 8 | [3 uninfected; 5 infected](https://github.com/hihisiv/phenodata/blob/main/GSE51436_d1.csv) | *Macaca mulatta* | 9544 |
| [GSE51615](https://www.ncbi.nlm.nih.gov/geo/query/acc.cgi?acc=GSE51615) | [GSE51615_d1](https://github.com/hihisiv/matrix_normalized/blob/main/GSE51615_d1-MatrixNormalized.csv) | uninfected | infected | GPL3535 | 23 | [9 uninfected; 14 infected](https://github.com/hihisiv/phenodata/blob/main/GSE51615_d1.csv) | *Macaca mulatta* | 9544 |
| [GSE51615](https://www.ncbi.nlm.nih.gov/geo/query/acc.cgi?acc=GSE51615) | [GSE51615_d2](https://github.com/hihisiv/matrix_normalized/blob/main/GSE51615_d2-MatrixNormalized.csv) | colon; uninfected | colon; infected | GPL3535 | 7 | [3 uninfected; 4 infected](https://github.com/hihisiv/phenodata/blob/main/GSE51615_d2.csv) | *Macaca mulatta* | 9544 |
| [GSE51615](https://www.ncbi.nlm.nih.gov/geo/query/acc.cgi?acc=GSE51615) | [GSE51615_d3](https://github.com/hihisiv/matrix_normalized/blob/main/GSE51615_d3-MatrixNormalized.csv) | jejunum; uninfected | jejunum; infected | GPL3535 | 8 | [3 uninfected; 5 infected](https://github.com/hihisiv/phenodata/blob/main/GSE51615_d3.csv) | *Macaca mulatta* | 9544 |
| [GSE51615](https://www.ncbi.nlm.nih.gov/geo/query/acc.cgi?acc=GSE51615) | [GSE51615_d4](https://github.com/hihisiv/matrix_normalized/blob/main/GSE51615_d4-MatrixNormalized.csv) | lung; uninfected | lung; infected | GPL3535 | 8 | [3 uninfected; 5 infected](https://github.com/hihisiv/phenodata/blob/main/GSE51615_d4.csv) | *Macaca mulatta* | 9544 |
| [GSE51615](https://www.ncbi.nlm.nih.gov/geo/query/acc.cgi?acc=GSE51615) | [GSE51615_d5](https://github.com/hihisiv/matrix_normalized/blob/main/GSE51615_d5-MatrixNormalized.csv) | colon; uninfected | jejunum; uninfected | GPL3535 | 6 | [3 colon uninfected; 3 jejunum uninfected](https://github.com/hihisiv/phenodata/blob/main/GSE51615_d5.csv) | *Macaca mulatta* | 9544 |
| [GSE51615](https://www.ncbi.nlm.nih.gov/geo/query/acc.cgi?acc=GSE51615) | [GSE51615_d6](https://github.com/hihisiv/matrix_normalized/blob/main/GSE51615_d6-MatrixNormalized.csv) | colon; uninfected | lung; uninfected | GPL3535 | 6 | [3 colon uninfected; 3 lung uninfected](https://github.com/hihisiv/phenodata/blob/main/GSE51615_d6.csv) | *Macaca mulatta* | 9544 |
| [GSE51615](https://www.ncbi.nlm.nih.gov/geo/query/acc.cgi?acc=GSE51615) | [GSE51615_d7](https://github.com/hihisiv/matrix_normalized/blob/main/GSE51615_d7-MatrixNormalized.csv) | jejunum; uninfected | lung; uninfected | GPL3535 | 6 | [3 jejunum uninfected; 3 lung uninfected](https://github.com/hihisiv/phenodata/blob/main/GSE51615_d7.csv) | *Macaca mulatta* | 9544 |
| [GSE51615](https://www.ncbi.nlm.nih.gov/geo/query/acc.cgi?acc=GSE51615) | [GSE51615_d8](https://github.com/hihisiv/matrix_normalized/blob/main/GSE51615_d8-MatrixNormalized.csv) | colon; infected | jejunum; infected | GPL3535 | 9 | [4 colon infected; 5 jejunum infected](https://github.com/hihisiv/phenodata/blob/main/GSE51615_d8.csv) | *Macaca mulatta* | 9544 |
| [GSE51615](https://www.ncbi.nlm.nih.gov/geo/query/acc.cgi?acc=GSE51615) | [GSE51615_d9](https://github.com/hihisiv/matrix_normalized/blob/main/GSE51615_d9-MatrixNormalized.csv) | colon; infected | lung; infected | GPL3535 | 9 | [4 colon infected; 5 lung infected](https://github.com/hihisiv/phenodata/blob/main/GSE51615_d9.csv) | *Macaca mulatta* | 9544 |
| [GSE51615](https://www.ncbi.nlm.nih.gov/geo/query/acc.cgi?acc=GSE51615) | [GSE51615_d10](https://github.com/hihisiv/matrix_normalized/blob/main/GSE51615_d10-MatrixNormalized.csv) | jejunum; infected | lung; infected | GPL3535 | 10 | [5 jejunum infected; 5 lung infected](https://github.com/hihisiv/phenodata/blob/main/GSE51615_d10.csv) | *Macaca mulatta* | 9544 |
| [GSE57730](https://www.ncbi.nlm.nih.gov/geo/query/acc.cgi?acc=GSE57730) | [GSE57730_d1](https://github.com/hihisiv/matrix_normalized/blob/main/GSE57730_d1-MatrixNormalized.csv) | non-progressor | HIV-1 progressor | GPL570 | 12 | [5 non-progressor; 7 progressor](https://github.com/hihisiv/phenodata/blob/main/GSE57730_d1.csv) | *Homo sapiens* | 9606 |
| [GSE61766](https://www.ncbi.nlm.nih.gov/geo/query/acc.cgi?acc=GSE61766) | [GSE61766_d1](https://github.com/hihisiv/matrix_normalized/blob/main/GSE61766_d1-MatrixNormalized.csv) | uninfected | acute infection | GPL3535 | 12 | [6 uninfected; 6 infected](https://github.com/hihisiv/phenodata/blob/main/GSE61766_d1.csv) | *Macaca mulatta* | 9544 |
| [GSE98717](https://www.ncbi.nlm.nih.gov/geo/query/acc.cgi?acc=GSE98717) | [GSE98717_d1](https://github.com/hihisiv/matrix_normalized/blob/main/GSE98717_d1-MatrixNormalized.csv) | uninfected | acute infection | GPL3535 | 8 | [4 uninfected; 4 acute](https://github.com/hihisiv/phenodata/blob/main/GSE98717_d1.csv) | *Macaca mulatta* | 9544 |
| [GSE98717](https://www.ncbi.nlm.nih.gov/geo/query/acc.cgi?acc=GSE98717) | [GSE98717_d2](https://github.com/hihisiv/matrix_normalized/blob/main/GSE98717_d2-MatrixNormalized.csv) | uninfected | chronic infection | GPL3535 | 7 | [4 uninfected; 3 chronic](https://github.com/hihisiv/phenodata/blob/main/GSE98717_d2.csv) | *Macaca mulatta* | 9544 |
| [GSE98717](https://www.ncbi.nlm.nih.gov/geo/query/acc.cgi?acc=GSE98717) | [GSE98717_d3](https://github.com/hihisiv/matrix_normalized/blob/main/GSE98717_d3-MatrixNormalized.csv) | acute infection | chronic infection | GPL3535 | 7 | [4 acute; 3 chronic](https://github.com/hihisiv/phenodata/blob/main/GSE98717_d3.csv) | *Macaca mulatta* | 9544 |
| [GSE119234](https://www.ncbi.nlm.nih.gov/geo/query/acc.cgi?acc=GSE119234) | [GSE119234_d1](https://github.com/hihisiv/matrix_normalized/blob/main/GSE119234_d1-MatrixNormalized.csv) | uninfected | infected | GPL21697 | 31 | [18 uninfected; 13 infected](https://github.com/hihisiv/phenodata/blob/main/GSE119234_d1.csv) | *Homo sapiens* | 9606 |
| [GSE119234](https://www.ncbi.nlm.nih.gov/geo/query/acc.cgi?acc=GSE119234) | [GSE119234_d2](https://github.com/hihisiv/matrix_normalized/blob/main/GSE119234_d2-MatrixNormalized.csv) | uninfected | B-cell germinal; infected | GPL21697 | 9 | [5 uninfected; 4 infected](https://github.com/hihisiv/phenodata/blob/main/GSE119234_d2.csv) | *Homo sapiens* | 9606 |
| [GSE119234](https://www.ncbi.nlm.nih.gov/geo/query/acc.cgi?acc=GSE119234) | [GSE119234_d3](https://github.com/hihisiv/matrix_normalized/blob/main/GSE119234_d3-MatrixNormalized.csv) | uninfected | B-cell unswitched; infected | GPL21697 | 9 | [5 uninfected; 4 infected](https://github.com/hihisiv/phenodata/blob/main/GSE119234_d3.csv) | *Homo sapiens* | 9606 |
| [GSE119234](https://www.ncbi.nlm.nih.gov/geo/query/acc.cgi?acc=GSE119234) | [GSE119234_d4](https://github.com/hihisiv/matrix_normalized/blob/main/GSE119234_d4-MatrixNormalized.csv) | uninfected | B-cell naive; infected | GPL21697 | 9 | [5 uninfected; 4 infected](https://github.com/hihisiv/phenodata/blob/main/GSE119234_d4.csv) | *Homo sapiens* | 9606 |
| [GSE119234](https://www.ncbi.nlm.nih.gov/geo/query/acc.cgi?acc=GSE119234) | [GSE119234_d5](https://github.com/hihisiv/matrix_normalized/blob/main/GSE119234_d5-MatrixNormalized.csv) | uninfected | B-cell memory; infected | GPL21697 | 9 | [5 uninfected; 4 infected](https://github.com/hihisiv/phenodata/blob/main/GSE119234_d5.csv) | *Homo sapiens* | 9606 |
