# Supplementary material for: HIHISIV: a database of gene expression in HIV and SIV host immune response
Source: BMC Bioinformatics. 2024 Mar 22;25:125. doi: 10.1186/s12859-024-05740-7 (PMC10958971; doi:10.1186/s12859-024-05740-7)
Supplement: Supplementary file 2 — Additional file 2 comprises the database design and description details. [file 12859_2024_5740_MOESM2_ESM.docx]

Supplementary Material 2: EXPERIMENT LIST

## Database, Design, and Description

To reproduce the concepts and relationships among entities such as genes, ontologies and experiments, we represent this scenario using a conceptual model (Figure S2.1) and the main entities are described below.

* **project**: describes information about the original project from the GEO. Contains db_accession (key), db_project_link, title, overall_design, and summary.

* **publication**: contains information about publication(s) related to the project. The attributes are pubmed_id , pubmed_link, doi, and title.

* **platform:** describes the kind of high-throughput gene expression platform used - RNA-Seq or microarray platforms. Contains information about GEO platform id (geo_platform_id), platform_link, platform_name, and annotation_platform.

* **experiment**: describes the experiments derived from the project (one or more). This entity contains information about the experiment_id (key), number of samples (n_samples), sample_description and observation.

* **experimental_factor_test**: contains attributes related to the group test such as the name factor used as comparisons (test_factor_name), the ontology name associated with the test_factor_name (test_factor_ontology_name), the ontology id and link, the species in the test group (test_sp), the tissue (test_tissue) and the ontology associated with this tissue (test_tissue_ontology), the virus lineage (in case of non-human) and the samples associated with this group.

* **experimental_reference_test**: similar to the entity experimental_factor_test but containing information on the reference group.

* **transcript**: it has a unique attribute describing the probe_id (key) related to the platform.

* **gene_symbol:** contains information about the gene and the attributes are: entrez_gene_id (key), gene_symbol (official HUGO name), link_ncbi, gene_name_desc, and chromosome.

* **deg_analysis***:* this entity contains the result of the DEG analysis by RNA-seq or microarray platforms. The attributes are experiment_id, transcript_id, log_fc values and adj_pvalue.

* **go_analysis**: this entity contains the results of the enrichment analysis. The attributes provided are gene ontology id (go_id), go_term, p-value, number of gene in the ontology id (no_go_size), and entrez_gene_id which represents the entrez_gene_id associated with this go_id in the result analysis.

* **orthologs**: this entity mapp the entrez_gene_id in *H. sapiens* to entrez_gene_id in *M. mulatta* (that is the common microarray platform used in the host of SIV experiments).


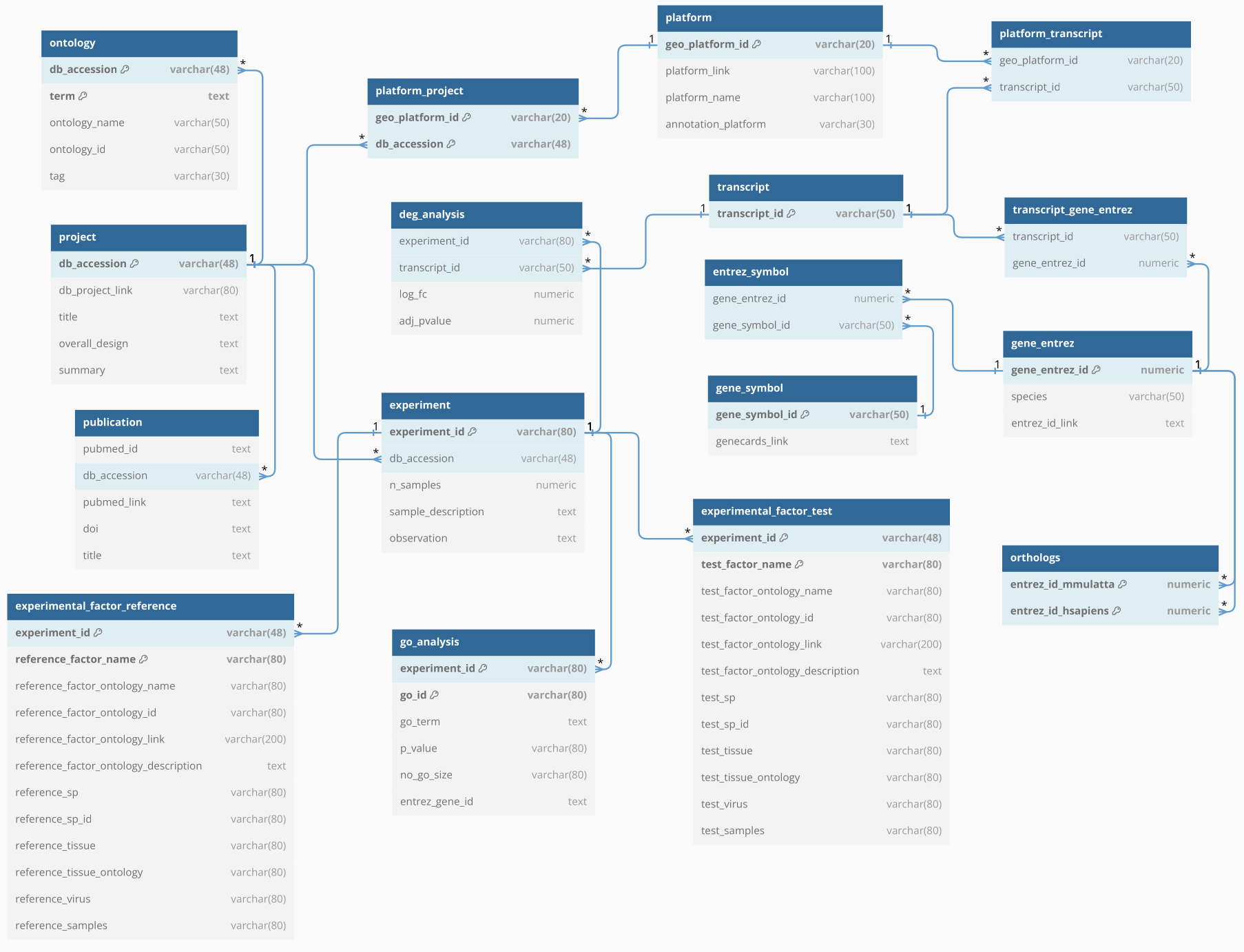


Figure S2.1: The HIHISIV conceptual model representing the database entities and their relationships. One of the main entities is ‘experiment’, which describes the experiments derived from the project (one or more).
